# Robotic Telemedicine for Mental Health: A Multimodal Approach to Improve Human-Robot Engagement

**DOI:** 10.3389/frobt.2021.618866

**Published:** 2021-03-18

**Authors:** Maria R. Lima, Maitreyee Wairagkar, Nirupama Natarajan, Sridhar Vaitheswaran, Ravi Vaidyanathan

**Affiliations:** ^1^ Department of Mechanical Engineering, Imperial College London, and UK Dementia Research Institute—Care Research and Technology Centre, London, United Kingdom; ^2^ Schizophrenia Research Foundation (SCARF), Chennai, India

**Keywords:** social robots, COVID-19, human-robot interaction, intelligent virtual assistant, multimodal interaction, dementia, telemedicine, low- and middle-income countries

## Abstract

COVID-19 has severely impacted mental health in vulnerable demographics, in particular older adults, who face unprecedented isolation. Consequences, while globally severe, are acutely pronounced in low- and middle-income countries (LMICs) confronting pronounced gaps in resources and clinician accessibility. Social robots are well-recognized for their potential to support mental health, yet user compliance (i.e., trust) demands seamless affective human-robot interactions; natural ‘human-like’ conversations are required in simple, inexpensive, deployable platforms. We present the design, development, and pilot testing of a multimodal robotic framework fusing verbal (contextual speech) and nonverbal (facial expressions) social cues, aimed to improve engagement in human-robot interaction and ultimately facilitate mental health telemedicine during and beyond the COVID-19 pandemic. We report the design optimization of a hybrid face robot, which combines digital facial expressions based on mathematical affect space mapping with static 3D facial features. We further introduce a contextual virtual assistant with integrated cloud-based AI coupled to the robot’s facial representation of emotions, such that the robot adapts its emotional response to users’ speech in real-time. Experiments with healthy participants demonstrate emotion recognition exceeding 90% for happy, tired, sad, angry, surprised and stern/disgusted robotic emotions. When separated, stern and disgusted are occasionally transposed (70%+ accuracy overall) but are easily distinguishable from other emotions. A qualitative user experience analysis indicates overall enthusiastic and engaging reception to human-robot multimodal interaction with the new framework. The robot has been modified to enable clinical telemedicine for cognitive engagement with older adults and people with dementia (PwD) in LMICs. The mechanically simple and low-cost social robot has been deployed in pilot tests to support older individuals and PwD at the Schizophrenia Research Foundation (SCARF) in Chennai, India. A procedure for deployment addressing challenges in cultural acceptance, end-user acclimatization and resource allocation is further introduced. Results indicate strong promise to stimulate human-robot psychosocial interaction through the hybrid-face robotic system. Future work is targeting deployment for telemedicine to mitigate the mental health impact of COVID-19 on older adults and PwD in both LMICs and higher income regions.

## Introduction

Dementia is a leading cause for disability and dependence across the world. As a chronic neurodegenerative condition, demands for care increase over time. Many people with dementia require social support, day care or assisted residence facilities with advancing illness. A staggering one in four United Kingdom hospital admissions is due to a dementia-related condition (Alzheimer's Research UK, 2020)^1^. Global care costs are projected to exceed $2 trillion/annum demanding 40 million new care workers, which could easily overwhelm medical and social care systems as they stand today (Alzheimer's Research UK, 2020)^1^. Prevalence of dementia is further skyrocketing in LMICs; 63% of PwD already live in LMICs, where 70% of new cases occur (Prince et al., 2007; Prince et al., 2013). In India, the treatment gap today is a staggering 90% (Dias and Patel, 2009). Lower-income nations will have comparable or even worse rates. Availability of resources, including human resource capacity, are major contributing factors to this gap (Prince et al., 2007; Shaji et al., 2010). Recent industrialization, migration, and urbanization in Asia have also impacted traditional family structures such that older people face less family support and more isolation today than ever before (Dias and Patel, 2009).

This global health crisis has become even more critical during the COVID-19 pandemic. However, planning and response to public health emergencies (i.e., COVID-19 outbreak) often do not directly address mental health, in particular for vulnerable groups such as older adults and PwD (Vaitheswaran et al., 2020). Dementia is already an emerging pandemic (Wang et al., 2020) with more than 50 million cases worldwide and a new case occurring every 3 s (Alzheimer’s Disease International, 2019)^2^. The combined strain of COVID-19 and dementia pandemics is severely increasing suffering of PwD and their caregivers. COVID-19 has caused unprecedented stress, fear and agitation among the seniors, especially those with cognitive impairment or dementia (Mehra and Grover, 2020), who are considered to be more vulnerable to COVID-19 (Wang et al., 2020). Isolation and confinement measures imposed to prevent infection of high-risk populations have undercut essential sources of support. Care is reduced or, in some cases, completely removed and important face-to-face contact lost, which may have long-lasting psychosocial and cognitive consequences in PwD. Caregivers for PwD are also in dire need of mental health support and many older adults without specific mental health diagnosis are also suffering pronounced psychological consequences due to isolation. Furthermore, the level of anxiety and exhaustion among staff in care residence facilities has increased during COVID-19 (Wang et al., 2020). There is immediate, urgent and desperate call for greater mental health support in this arena.

The need for mental health and psychosocial support of PwD and their carers worldwide is well-documented (Wang et al., 2020) both during and prior to the COVID-19 pandemic. The effect of COVID-19 on healthcare infrastructure in LMICs, however, is arguably more extreme due to the health system capacity and deeper dependency on families for support of PwD (Walker et al., 2020). A recent study conducted with caregivers in South India (Vaitheswaran et al., 2020) highlights a clear need for more services and support of PwD and caregivers for the post-pandemic, including stronger adoption of technology. Affordable, accessible and scalable solutions to monitor mental health, improve independence, increase quality of life, and reduce caregiver burden are urgently needed both in the immediate situation, as well as beyond the current global lockdowns.

Socially assistive robots (SAR) are well-documented for promise to support dementia and mental health (Tapus et al., 2007), with strong potential specifically to mitigate COVID-19 impact on PwD. Prior to the COVID-19 pandemic, a range of tools from simple voice interfaces to interactive social robots have been introduced with the aim of providing stimulation, entertainment, personal assistance, monitoring and safety for older adults and PwD (Inoue et al., 2012; Martín et al., 2013; Mordoch et al., 2013; Joranson et al., 2015; Moyle et al., 2017; Falck et al., 2020); see (Abdi et al., 2018) for a recent review. Exemplary cases such as the humanoid robot NAO (Agüera-Ortiz et al., 2015), PaPeRo (Inoue et al., 2012), Bandit (Tapus et al., 2009), Eva (Cruz-Sandoval and Favela, 2016), and robot alternatives to animal assisted therapy such as AIBO, the robotic dog (Tamura et al., 2004), NeCoRo, the robotic cat (Libin and Cohen-Mansfield, 2004), and the well-known Paro, the robotic seal (Wada and Shibata, 2007) have shown the possibility of improving patient engagement, reducing agitation, improving mood and communication, and decreasing stress (Inoue et al., 2011; Petersen et al., 2017), though comparable results have been argued with a simple stuffed animal (Moyle et al., 2017). Recent literature (Martín et al., 2013; Valenti Soler et al., 2015; Rouaix et al., 2017) has argued social robots can help improve irritability, global neuropsychiatric symptoms, and PwD’s emotional responses with robot assisted therapies. The neuropsychological effects of interaction with robots has also shown increased cortical neuron activity (Wada et al., 2005a). Social robots hold specific promise in the COVID-19 crisis by providing older adults and PwD with complementary support to alleviate anxiety and loneliness, improve engagement, and reduce caregiver burden. Social robots can also provide clinicians with an alternative platform to deliver remote therapies and support PwD, especially at a time when nonemergency clinical appointments are increasingly shifting to remote alternatives.

There are several verbal and nonverbal interaction modes used by SAR to engage with humans—such as facial expressions, speech, gestures, or behavior—but the most effective communication mode in human-robot interaction (HRI) is largely considered to be speech (Fong et al., 2003). Intelligent virtual assistants (IVA), also known as conversational agents, chatbots, or virtual assistants, are AI-powered systems that understand user intents in natural language and generate relevant responses using machine learning (ML) algorithms. Despite benefits in feasibility of implementation and commercial availability, virtual assistants and voice-enabled smart speakers (e.g., Amazon Alexa, Google Home) alone have limited flexibility to adapt for mental health care applications, particularly to support PwD. Emerging social robots, on the contrary, can use a multimodal approach with combined social and emotional nonverbal cues, such as facial expressions or gestures, in addition to verbal communication. The integration of affective communicative modalities and implementation in social robots can ultimately lead to improved engagement in HRI in demanding healthcare contexts, such as dementia care, during and beyond the COVID-19 pandemic. Social robots can be used as a means of telemedicine and remote therapy delivery for mental health monitoring and psychosocial support of high-risk populations, particularly older adults and PwD. By providing companionship and enhancing independent living, such robotic technologies could also give carers some respite time and relieve the caregiver burden especially faced during lockdowns.

The high cost of such complex interactive systems is oftentimes a barrier for deployment, especially in LMICs. While SAR have shown great potential to support PwD in clinical and residential environments, no research to date has developed and pilot tested mechanically simple and low-cost robotic solutions to support dementia care in challenging scenarios imposed by the COVID-19 outbreak in LMICs, particularly in India.

The goal of this investigation is to design, develop, and test the feasibility of a social robotic platform to support people with dementia, during and following the COVID-19 pandemic. We are particularly interested in surmounting cost constraints and ease of use demands for utility in LMICs. In our previous work (Bazo et al., 2010) we introduced a ‘hybrid face’ robot with combined static physical features and a digital face that simulates facial expressions based on mathematical affect space emotion mapping. We further quantified the neurophysiological response to the robot and initiated work to translate a simplified version of it as a consumer product (Wairagkar et al., 2020). In this study, we:

(1) Propose a new multimodal robotic framework that integrates animated representation of emotions with a voice system supported by a cloud-based AI conversational engine for enhanced engagement in HRI.
(2) Test human-robot multimodal interactions with healthy participants in the United Kingdom demonstrating the robot’s ability to adapt facial expressions to users’ speech in real-time and a strong user preference for multimodal vs. pure voice communication.
(3) Introduce a user-centred procedure for cultural validation addressing telemedicine acceptance of robotic mental health support of older individuals and PwD. Modifications to our robotic platform based on this procedure as implemented in South India are presented, and the procedure is offered as a broader method for introduction of such technology in new cultural arenas and demographics.
(4) Present a pilot study introducing the robot into practice for dementia support in LMICs through experiments conducted with people with dementia at the Schizophrenia Research Foundation (SCARF) in Chennai, India.

Results demonstrate the capacity to deliver telemedicine cognitive engagement and mental health support through the hybrid face robot. Current work is targeting trials in South India with planned investigations on deployment in LMICs as well as wealthier nations.

## Related Work

The ability to recognize, understand, and show emotions plays a fundamental role in the development of SAR capable of meaningful interactions (Breazeal, 2009). Facial expressions, speech, and body language are proved to carry essential affective information for social interactions (Breazeal, 2003). According to (Schiano et al., 2000) facial expressions are the primary means of communicating emotions. Ekman introduced the facial action coding system (FACS) (Donato et al., 1999) and posits that all human expressions are a combination of the primary expressions: happiness, sadness, anger, fear, disgust, and surprise.

Robotics researchers are faced with the question of whether to design physically embodied, fully actuated robots, or simpler and cheaper virtual agents. Literature has argued the level of a robot’s embodiment is key to develop trust and rapport, and may affect human judgements of the robot as a social partner (Wainer et al., 2006; Bainbridge et al., 2011). (Ghazali et al., 2018) have suggested trust toward robotic agents is also influenced by its facial characteristics. Though it remains unclear how robot’s gender shapes human trust, in this experimental study, gender did not affect user trust and a higher psychological reactance was observed in participants during interactions with a robot of opposite gender. Embodiment is also an influencing factor of users’ expectations of the robot’s abilities and autonomy (Clabaugh and Matarić, 2019). However, the mechatronic complexity in the development of embodied, fully actuated robots with the desired expressive ability is associated with high costs. This in turn constitutes a barrier for real-world deployment beyond academic research, especially in LMICs. The implementation of expressive robotic faces on LCD screens has recently been applied in different SAR platforms, which allows easy customization, adaptability to users’ preferences and culture, higher accessibility and scalability (Abdollahi et al., 2017; Portugal et al., 2019); this may be especially relevant for human-robot engagement with older adults with and without dementia, as the screen can also be used for interactive activities, visualization of the robot’s speech, or as an additional user input. Regardless of the social robot level of embodiment, facial characteristics, or gender, care should be taken to avoid reaching the uncanny valley, graphically represented by a sudden negative drop in human’s emotional response toward robots when shifting from non-human/artificial faces toward optimal human faces (Mori et al., 2012). Additionally, when designing social robots to effectively interact with older citizens and cognitive impaired individuals, researchers must consider ethical concerns that may limit the deployment of such technologies. These include increased use by vulnerable populations, reduced human contact, loss of privacy, emotional deception, which occurs when users’ expectations of the robot are not met, and attachment to the robot, which may cause emotional distress (Van Maris et al., 2020; Van Patten et al., 2020). In a longitudinal study with older citizens, (Van Maris et al., 2020) highlight the need for research metrics to analyze emotional attachment to social robots, people’s behaviors, and speech patterns.

The field of ML has recently experienced extraordinary progress in the development of IVA. These AI-powered systems interact with users using natural language and are able to generate relevant responses in the form of text, speech, or both. This technology started in the 1960s with ELIZA (Weizenbaum, 1966), which held text-based conversations with users acting as a psychotherapist. A recent body of work (Romero et al., 2017; Harms et al., 2018) has provided novel approaches for the development of conversational agents for increased user engagement. Along similar lines, there has been work on the development of embodied conversational agents—virtual animated characters, usually with the appearance of a human-like avatar, capable of understanding multimodal utterances, such as voice, gestures, and emotion (Griol et al., 2019). These systems aim to provide a more empathetic response based on dialogue and behavior (Merdivan et al., 2019), yet they often cause a sense of discomfort explained by the uncanny valley (Ciechanowski et al., 2019).

Intelligent virtual assistants have been deployed in healthcare for delivering cognitive behavior therapy (Fitzpatrick et al., 2017) or assist older people in the living environment; see (Laranjo et al., 2018) for a recent review. Yet, evidence of efficacy and safety of conversational agents to reliably support healthcare is limited (Laranjo et al., 2018); prior research has reported inconsistent responses even when user statements explicitly contained risk or harm (e.g., “I want to commit suicide,” “I am depressed”) (Miner et al., 2016). To assist those with cognitive impairments, (Wolters et al., 2016) have explored the use of virtual assistants with PwD, highlighting the importance of adapting voice and interaction style to each user’s preferences and expectations, but importantly, to cognitive decline. One interesting observation that deserves more introspection is that people with dementia questioned the acceptability of a voice system without a face. Furthermore, the COVID-19 outbreak has spurred greater interest in the use of voice assistants and chatbots as a tool to support high-risk populations, such as older individuals and PwD; if designed effectively, these may support patients in need for routine care via conversations, provide up-to-date information, and alleviate the mental health burden (Miner et al., 2020; Sezgin et al., 2020).

## Methods

### Development of Multimodal Robotic System

#### Affective Hybrid Face Robot

**FIGURE 1:**
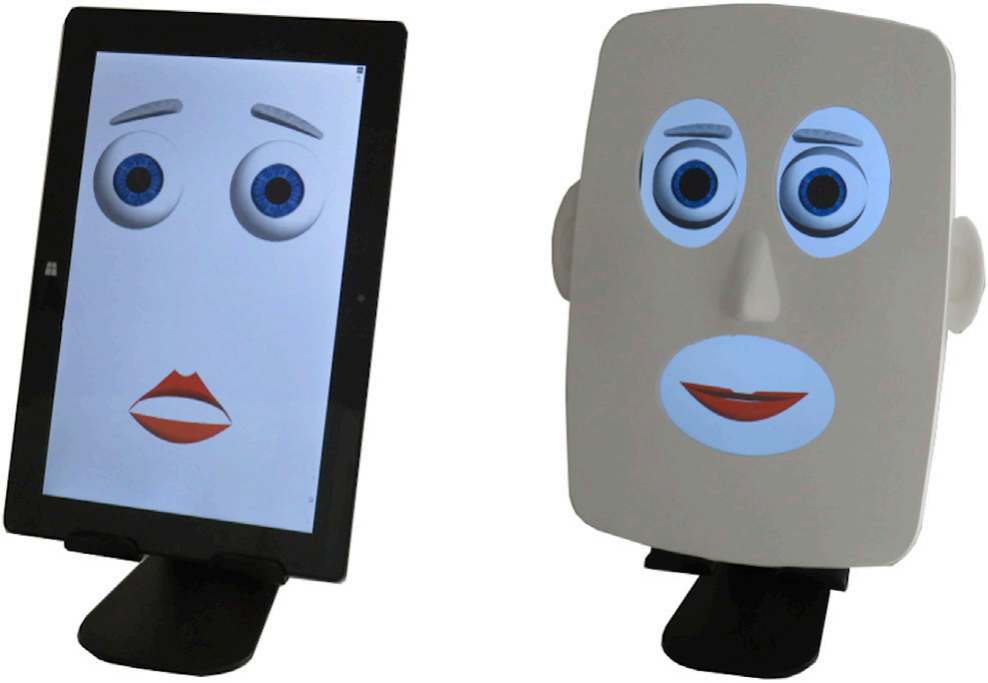
Hybrid face robot running on a tablet PC with option to add a 3D printed faceplate for realism and depth.

The hybrid face robot integrates a digital face capable of showing facial expressions and a 3D printed faceplace to convey realism and depth, which can be flexibly added to the robot (Figure 1). The robot’s software was programmed using Max 8 (Cycling ’74, San Francisco, CA, United States) and was implemented on a tablet PC, building upon our previous work (Bazo et al., 2010).

**FIGURE 2:**
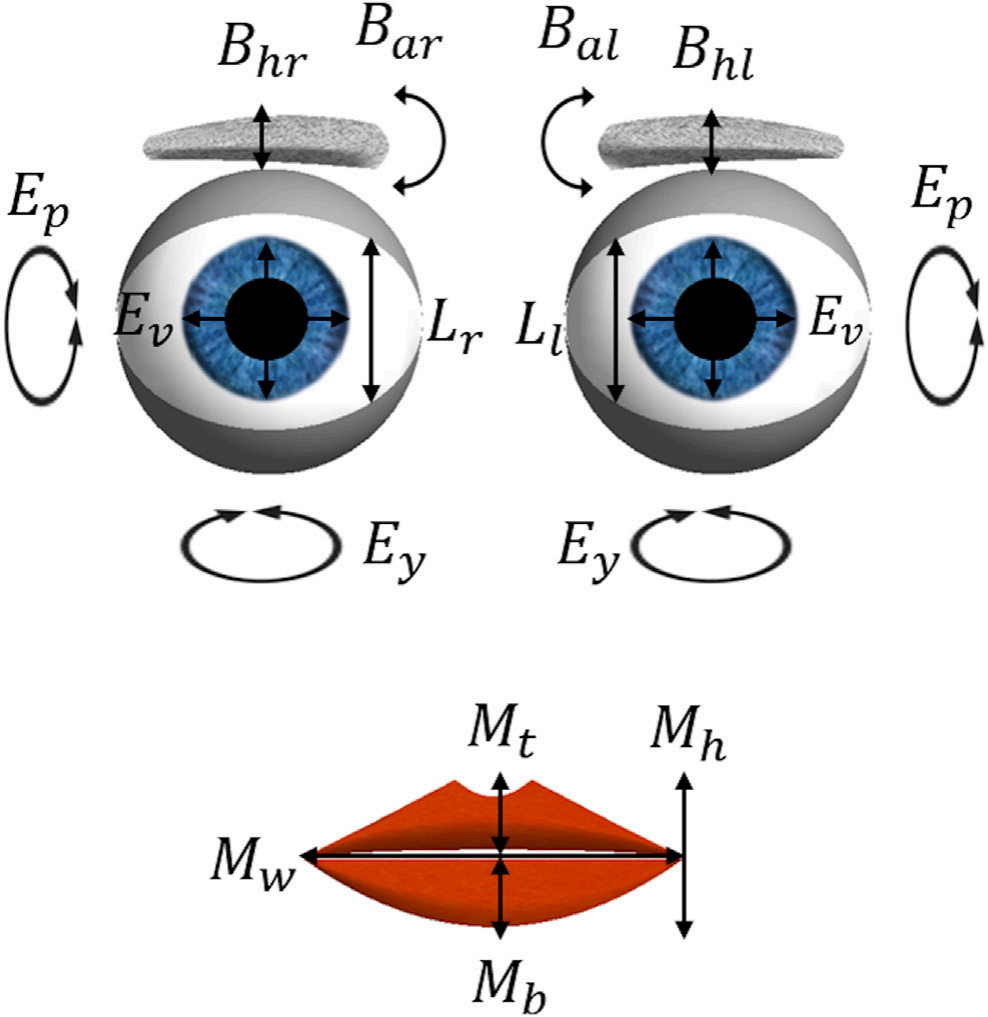
The 13 degrees of freedom of the expressive face: eyebrow angles (B_al_ and B_ar_) and vertical height (B_hl_ and B_hr_); eyelid openness (L_l_ and L_r_); eye vergence (E_v_), pitch (E_p_) and yaw (E_y_); mouth corner vertical height (M_h_), width (M_w_), top lip openness (M_t_) and bottom lip openness (M_b_).

The robotic face is simply made of four facial features: eyebrows, eyelids, eyes, and lips, with a total of 13 degrees of freedom (DoF) illustrated in Figure 2. Realism features, such as constant motion of the face, random blinking of the eyes, and pupil dilation (Bazo et al., 2010; Craig et al., 2010; Wairagkar et al., 2020) can be controlled and may lead to more dynamic HRIs. Our choice of a simplistic three-dimensional design for the robotic face aims to avoid the uncanny valley effect. Ideally, this mechanically simple robot would elicit human-like trust and engagement in HRIs, yet without the mechatronic complexity and associated high-costs of a fully actuated face.

The robot’s software design is based on a mathematical approach for emotion mapping, in which the robot’s expression state, $\vec{e}\left(t\right)$, for any given time, *t*, is defined as the weighted linear combination of a set of basis expressions $B=\left\{{\vec{b}}_{1},{\vec{b}}_{2},\ldots ,{\vec{b}}_{n}\right\}$. Each vector contains 13 values, one for each degree of freedom of the digital face. In our previous work (Bazo et al., 2010), this set has been defined with the following expressions: $B=\left\{{\vec{b}}_{\mathrm{happy}},{\vec{b}}_{\mathrm{sad}},{\vec{b}}_{\mathrm{angry}},{\vec{b}}_{\mathrm{stern}},{\vec{b}}_{\mathrm{surprised}},{\vec{b}}_{\mathrm{disgusted}},{\vec{b}}_{\mathrm{afraid}},{\vec{b}}_{\mathrm{tired}}\right\}$. The intensity vector $\vec{w}={\left[w_{1},w_{2},\ldots ,w_{n}\right]}^{T}$, $w_{i}\in \left[0,1\right]$, symbolizes the amount by which an expression ${\vec{b}}_{i}$ contributes to $\vec{e}\left(t\right)$. Hence, any expression state is the weighted sum of variances of each basis expression, ${\vec{b}}_{i}$, from the neutral expression, ${\vec{b}}_{N}$, and then added to the neutral expression along the following equation (time is omitted from notation for simplicity):

$$\vec{e}=\sum_{i=1}^{n}\left({\vec{b}}_{i}-{\vec{b}}_{N}\right){\vec{w}}_{i}+{\vec{b}}_{N}$$ (1)

**FIGURE 3:**
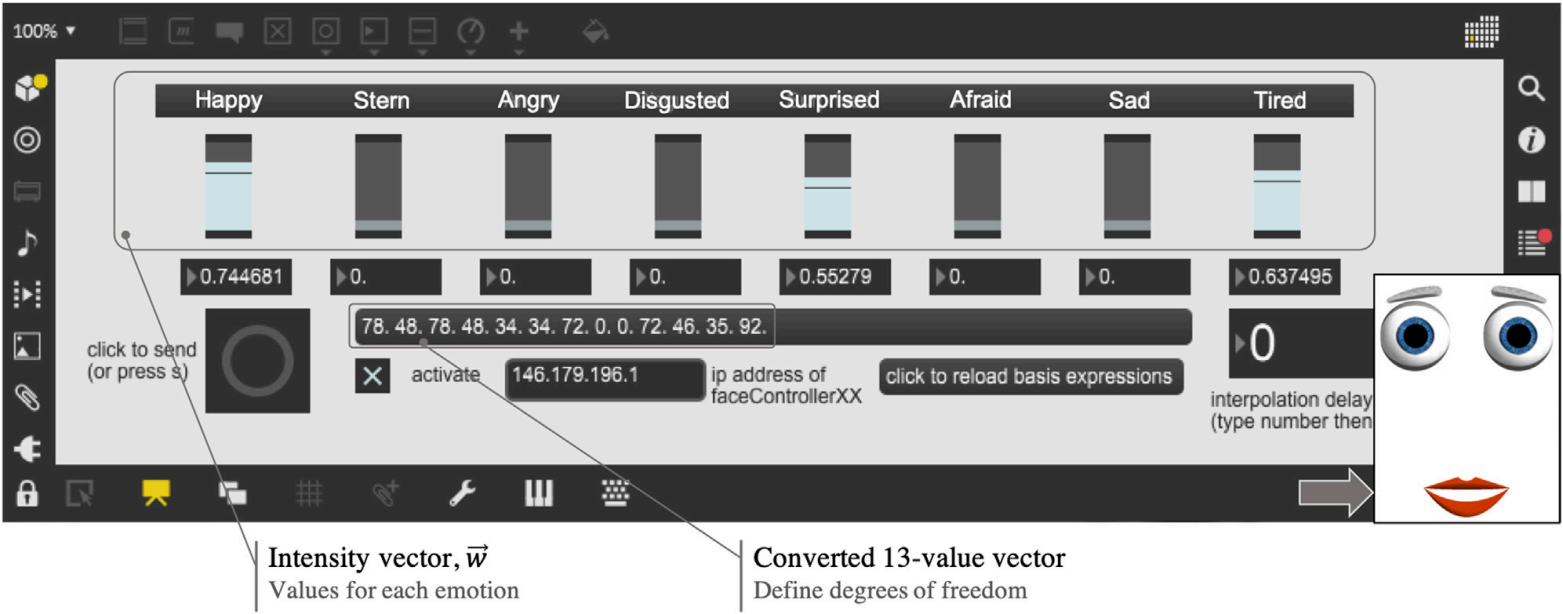
Front-end control of robotic animated facial expressions.

Following this approach, the modeling of emotions can be manually and remotely controlled by selecting each expression’s intensity, which is in turn converted into a 13-value vector, defining the DoF of the desired facial expression (Figure 3). Additionally, the robot’s mouth is animated and synchronized with the audio input’s amplitude, in decibel (dB), in such a way that when it surpasses a cut-off dB value the DoF correspondent to the top and bottom lips (M_t_ and M_b_, respectively) open and close simultaneously, while the mouth width, M_w_, changes by 30% to simulate the elastic movement of a human mouth.

The design of robot’s facial expressions and its control system were optimized in this work. This optimization included: 1) creating an interface with Python to enable automatic and remote control of facial expressions integrated with autonomous speech (see Section *Intelligent Virtual Assistant*); 2) optimizing the voice stream synchronization parameters in Max software; 3) modifying DoF for some expressions to improve recognition rates. Taken together, these improvements allowed us to:

(1) Integrate the robot’s speech capacity with facial expressions for adaptable emotion in response to users’ speech in real-time.
(2) Introduce the robot into practice for aging and dementia support in LMICs; a pilot study was conducted at SCARF India using the hybrid face robot as a telemedicine tool for remote cognitive engagement with a person with dementia through repeated sessions, in the form of Wizard of Oz experiment [see (Natarajan et al., 2019) for our recent work].

Our aim is to enhance engagement in HRI by endowing the robot with a set of multimodal affective cues (i.e., verbal and nonverbal, through facial expressions), and further validate the cultural acceptability and usefulness of such robotic tool as a telemedicine solution for mental health support of older adults and people with dementia in India. This may be of special interest (but not limited to) in the context of COVID-19, particularly in LMICs.

**FIGURE 4:**
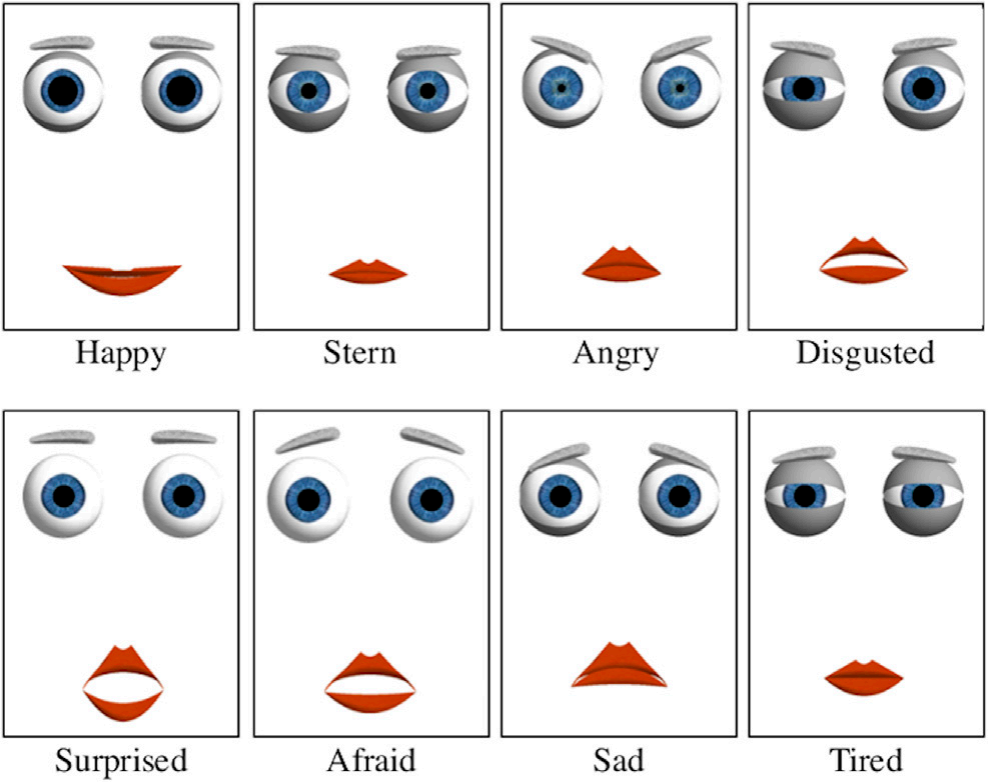
Optimized design of facial expressions simulated by the robot. Major modifications were made for the disgusted, surprised, and afraid robotic expressions with the aim of improving emotion recognition rates.

Figure 4 shows the optimized design of different facial expressions simulated by the robot. Importantly, the disgusted expression, which as pointed by Ekman in (Donato et al., 1999) features a peculiar wrinkling of the nose impossible to simulate with the current face design, was redesigned following psychology and robotics literature (Donato et al., 1999; Breazeal, 2003): the eyes were narrowed, by decreasing L_l_ and L_r_ and adjusting the eyelid asymmetry; eyebrows were lowered, with a significant change in the right angle, B_al_; upper and lower lips were raised, and the mouth was slightly opened, by adjusting M_t_ and M_b_; lastly, the mouth width, M_w_, was increased. Afraid and surprised expressions were modified to avoid past confusion between one another (Bazo et al., 2010), as both feature raised eyebrows, opened eyes and mouth. Major modifications included: for surprised, the DoF M_t_ and M_b_ were increased to their maximum values and M_w_ decreased to the minimum; for afraid, the eyebrows vertical height was raised (DoF B_hl_ and B_hr_) and the angles were slightly rotated (DoF B_al_ and B_ar_). Other expressions were used as in previous work (Bazo et al., 2010; Craig et al., 2010).

#### Intelligent Virtual Assistant

In order to extend the social robot’s autonomous interactive capabilities and explore multimodal affective HRI, we developed a virtual assistant powered by state-of-the-art IBM Watson (IBM, New York, United States) cloud-based AI capabilities. These AI cloud services have been used in past research in robotics and computer science (Chozas et al., 2017; Novoa et al., 2018; Di Nuovo et al., 2019). Overall, the implementation of the multimodal architecture described below allows the robot to emotionally interact with humans through voice, in addition to simulated facial expressions, and adapt the displayed emotion depending on users’ speech in real-time.

##### System Architecture

The system uses speech recognition algorithms, natural language understanding (NLU), and the training data provided to simulate a natural conversation. The cloud-based AI conversational system was designed to interact with users through speech, text, or both, maintaining a conversation in four different domains of knowledge, i.e., skills, atomic programs that represent a capability in a specific domain. The implemented skills enhance the flow of conversation and lead the system to:

• Converse about the user’s emotional state.
• Entertain the user with a quiz on selected topics.
• Provide definitions of any concept the user asks about (integration with Wikipedia^3^).
• Give local weather forecasts if requested (integration with The Weather Company^4^).

**FIGURE 5:**
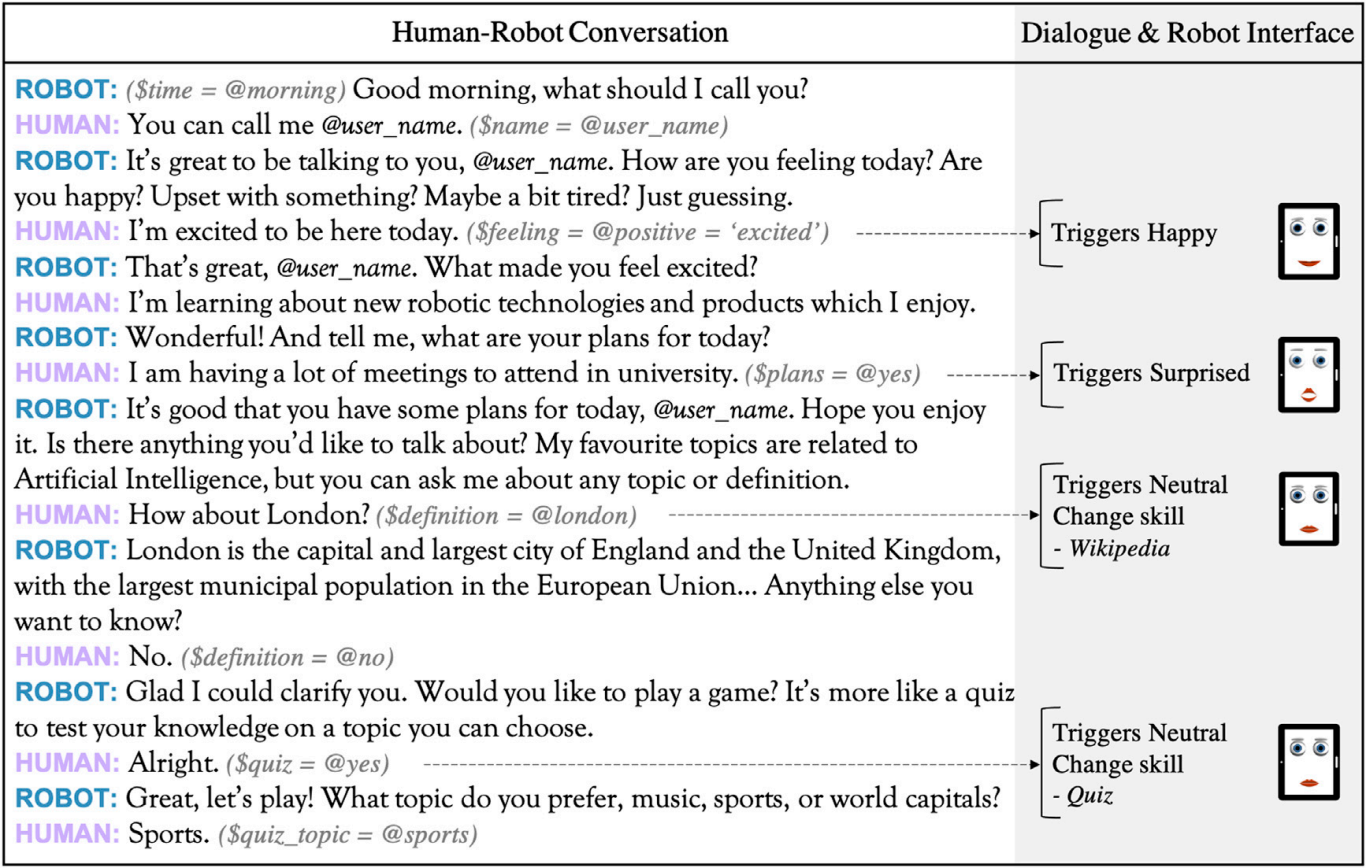
Section of a human-robot conversation transcript from testing with healthy participants in the United Kingdom. Various context variables ($) and entities (@) are identified. These allow to 1) change dialogue skills (e.g., gather definitions from Wikipedia, start a quiz to entertain the user), and 2) simultaneously adapt the robot’s facial representation of emotion according to the sentiment in user response, ultimately leading to more engaging and personalized interactions.

To create natural, believable interactions between intelligent virtual assistants and humans, understanding the context of conversations is of utmost importance (Harms et al., 2018). Therefore, for each dialogue skill designed several context variables were programmed (i.e., information that is stored during the dialogue), such as the user’s name, mood, time of day, or location. These allowed a certain degree of personalization, in that for each interaction the system dynamically tailors responses to user preferences and mood. Figure 5 shows an extract of a human-robot conversation, including different dialogue skills, context variables and the interface with the affective hybrid face robot (see Section *Implementation* for further details). Our principal aim in this investigation was to integrate the IVA system with the robot’s affective framework and address feasibility of acceptance and deployment. In future work we intend to introduce a knowledge base with user profiles and implement machine learning to personalize interactions over time.

##### Implementation

Several APIs were programmable combined and integrated with the robot’s affective capabilities in the back-end system by an orchestrator coded in Python. The main cloud-based services used include: 1) IBM Watson Assistant to create a dialogue flow, context variables, and provide training data—intents and entities, the user goal and its context, respectively; 2) IBM Tone Analyzer, which detects sentiment from text; 3) Google Speech to Text to perform speech recognition; 4) Google Text to Speech to generate the robot’s voice with a relevant response.

The interface created in Python between the hybrid-face robot and the IVA takes as input 13-integer strings via sockets UDP and local IP address, which represent the 13 degrees of freedom for each facial expression simulated by the robot. The default facial expression simulated by the robot was defined as happy. The orchestrator is therefore responsible to manage the flow of conversation by 1) controlling the jump between several dialogue skills and 2) adapting the robot’s facial expression depending on users’ responses in real-time, which are analyzed by IBM Tone Analyzer and sent to the robot’s software (e.g., if the user says “I had a bad day” the robot will verbally reply while displaying a sad expression). To trigger a conversation ‘skill’ and subsequently a dialogue node, the IVA algorithm evaluates intents, entities, and context variables included in the user response. This process is done based on the confidence level, i.e., the probability that the variable was correctly identified, with regards to the examples given when training the algorithm. The confidence scoring (decimal in the range 0–1) is done independently of previous utterances and its default threshold is 0.3^5^.

**FIGURE 6:**
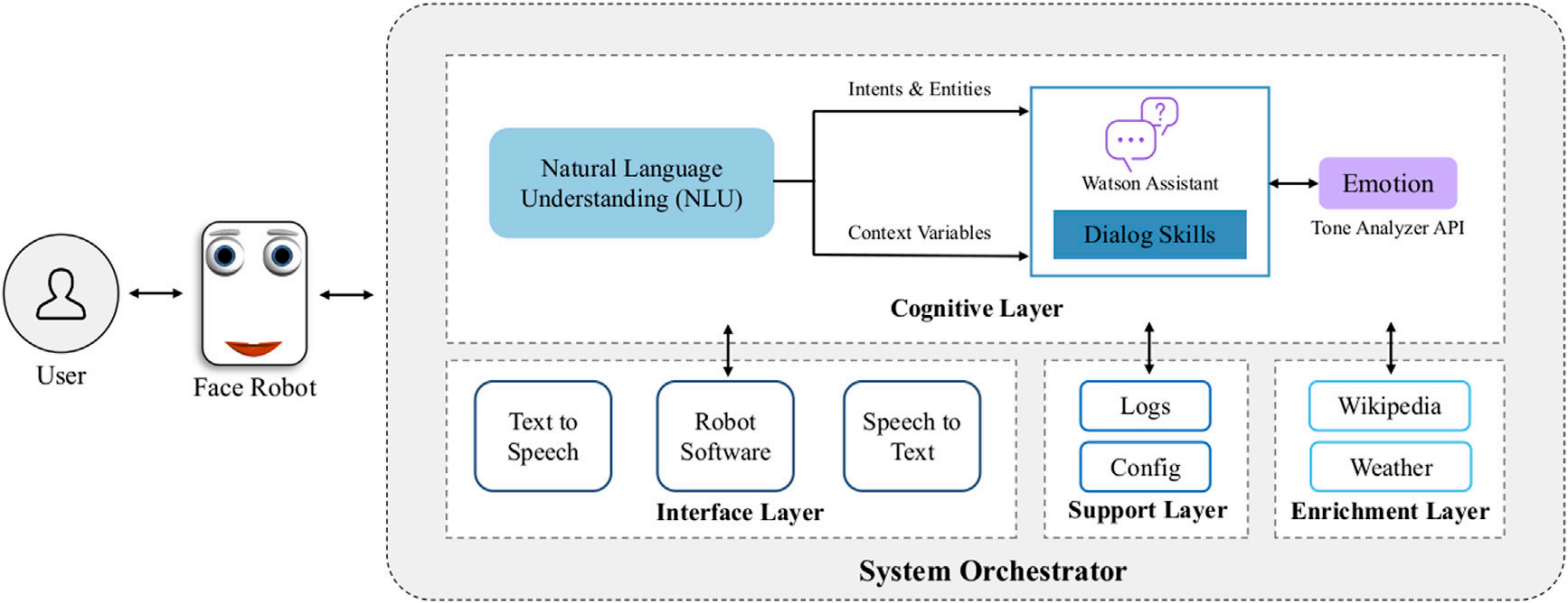
Proposed architecture design of the intelligent virtual assistant for engagement in multimodal HRI. Multiple cloud-based AI services were combined and integrated with the robot’s affective capabilities in the back-end system by an orchestrator coded in Python. The orchestrator manages the flow of conversation and adapts the robot’s facial representation of emotion in response to users’ speech in real-time. The orchestrator coordinates the interface, cognitive, support and enrichment layers.

Figure 6 illustrates the four layers coordinated by the orchestrator, which were designedly integrated to enhance engagement in multimodal HRIs. The *interface layer* includes APIs for communication (speech and text), the interface with the robot’s software and control of facial expressions; it receives input from the *cognitive layer*, which comprises the cognitive process of understanding user inputs through NLU. This is algorithmically achieved by recognizing intents, entities and context variables, and by analyzing the emotion in user responses. Further, the *enrichment layer* establishes communication with external services to get information about the current weather (The Weather Company and Python library geopy were used to locate coordinates and provide weather forecasts), or general definitions provided by Wikipedia. Lastly, the *support layer* is responsible for all links with external services and APIs, i.e., config files, and stores all the cognitive processes involved in interactions, indicating intents, entities and context variables recognized for each user response, their confidence level, or possible errors encountered (i.e., application logs). The latter were utilized for algorithm training using the Watson Assistant platform so that the system could understand different natural language syntaxes, adapt its responses, and retrain itself in case the wrong intent was identified.

### Evaluation of Human-Robot Interactions with Healthy Participants

In order to evaluate interactions with the multimodal robotic system proposed, we conducted a user study to assess emotion recognition of simulated facial expressions and the user experience in human-robot multimodal interactions. Ethics clearance was obtained by Imperial College London Science, Engineering and Technology Research Ethics Committee (SETREC). Written informed consent was obtained from participants.

#### Emotion Recognition Experiments

Recognition of Ekman’s basic expressions is a standard test to assess the emotional abilities of an expressive robotic face (Schiano et al., 2000; Breazeal, 2003). Therefore, an expression recognition task was conducted with N = 15 healthy participants (23–49 years, 3 female, 12 male) in the United Kingdom to qualitatively assess the optimized design (Section *Affective Hybrid Face Robot*) of the affective robotic face, particularly the disgusted, afraid and surprised facial expressions. Participants were given a list with the eight facial expressions and were shown a sequence of the robot’s eight expressions (see Figure 4) of approximately 5 s each. After each facial expression observed, participants chose the best match from the given list, following a forced-choice paradigm in line with research (Breazeal, 2003). The hybrid face robot was shown both with and without the 3D faceplate (see Figure 1) to address its likeability. After the task, qualitative feedback was gathered from in-depth interviews to understand how the design of robot’s facial expressions could be adjusted for further experiments and address the overall impression of the robot.

#### User Experience Experiments

A user experience questionnaire (UEQ) was used to evaluate the multimodal robotic system. The UEQ matched those deployed for measuring the user experience of interactive products (Schrepp, 2015; Memeti and Pllana, 2018). Our testing was completed with N = 10 healthy volunteers (21–59 years, 5 female, 5 male) who had never interacted with the hybrid face robot but had previous experience with interactive technologies. This study was implemented as a new experiment drawing on findings from the emotion recognition task (Section *Emotion Recognition Experiments*). It was conducted independently with a different set of users to obtain entirely unbiased user experience feedback. For instance, the additional 3D faceplate was not used in this study as it was not perceived favourably in previous human-robot interaction experiments, suggesting a potential uncanny valley effect, induced when the faceplate was added to the digital face (see Section *Emotion Recognition*). The main goal of the UEQ is to evaluate the interaction and engagement between participants and the robotic system. The UEQ used in this study considers five aspects: *attractiveness* evaluates the overall impression of the robot; *perspicuity* assesses the difficulty level to get familiar with the robotic tool; *efficiency* relates to the effort required to understand the robot’s emotional responses; *stimulation* evaluates how motivating and exciting is to interact with the robot; lastly, *novelty* judges how innovative and creative the robotic system was perceived by users.

Participants were seated in front of the robot and interacted with it, through speech and visualization of simulated facial expressions. The IVA system was activated in one laptop, and a speaker was placed behind the tablet PC, where the robot’s software runs. This allowed a better synchronization of the robot’s mouth animation and the audio signal (dB), in such a way that the robot is assumed to be the one speaking. Following human-robot interactions, participants answered the UEQ. Table 1 lists the questions used for each aspect in this UEQ analysis. The Likert scale system (Boone and Boone, 2012) was used in this method with a scale range from 1 to 5 (1 represents the most negative answer, 3 a neutral answer, and five the most positive answer). For the novelty aspect, participants were asked to choose between two terms with opposite meaning, using the same scale. Afterward, we conducted a short in-depth interview with the aim of qualitatively understanding benefits of multimodal vs. pure face or voice interactions. Specifically, participants were asked whether they would prefer to verbally interact with the virtual assistant (voice only), or with the multimodal robotic system instead (speech integrated with facial expressions).

**TABLE 1:** Questions selected for the user experience questionnaire (UEQ) evaluating response to the multimodal robotic system. Questions were grouped to evaluate five aspects: attractiveness evaluates the overall impression of the robot; perspicuity assesses the difficulty level to get familiar with the robotic tool; efficiency addresses the effort required to understand the robot’s emotional responses; stimulation judges how motivating and exciting human-robot interactions are perceived; novelty relates to how innovative and creative the robot was perceived by end-users.

Aspect	Id	Question
Attractiveness	a1	What is your overall impression of the proposed robotic system?
	a2	How useful do you find the possibility to communicate with voice?
	a3	How attractive and friendly do you find the robot’s facial expressions?
Perspicuity	p1	How intuitive are the robot’s emotions?
	p2	How clear are the robot’s responses?
	p3	How easy is it to communicate with the robotic system?
Efficiency	e1	How efficient is the robot to convey emotion through speech and expressive faces?
	e2	How practical are the robot’s answers or suggestions?
Stimulation	s1	How exciting is to communicate with this robotic system?
	s2	How interesting was the conversation/interaction?
	s3	How much does the robot motivate you to have new interactions?
Novelty	n1	Dull/creative
	n2	Conventional/Inventive
	n3	Usual/leading edge
	n4	Conservative/innovative

### Robotic Telemedicine for Mental Health Support

The overarching goal in this study is to facilitate introduction of the robot into practice for mental health and PwD support in LMICs. We introduce a user-centred procedure for cultural adaptation of the robot in the context of South India and describe the infrastructure to deploy it as a telemedicine tool to deliver regular cognitive engagement. The pilot study here described is the first of its nature to explore the feasibility and cultural acceptability of robotic telemedicine for mental health and dementia support in India. This may be of particular interest during and following the COVID-19 context to alleviate end-user anxiety and loneliness, improve engagement, and reduce the caregiver burden especially faced during lockdowns.

#### Designing Infrastructure

Due to regional language barriers, the AI voice system integration (Section *Intelligent Virtual Assistant*) was not suitable for clinical experiments conducted at SCARF India. Hence, we designed a test infrastructure to use the hybrid face robot as an assistive tool to deliver meaningful cognitive engagement with PwD and healthy older adults in Chennai, India. This infrastructure gives medical professionals the remote control of robot’s animated facial expressions and speech. The proposed experimental approach may provide clinicians with an alternative platform for remote therapy delivery and enhanced mental health support of PwD, in particular that meets the cost constraints and ease of use demands for utility in LMICs.

**FIGURE 7:**
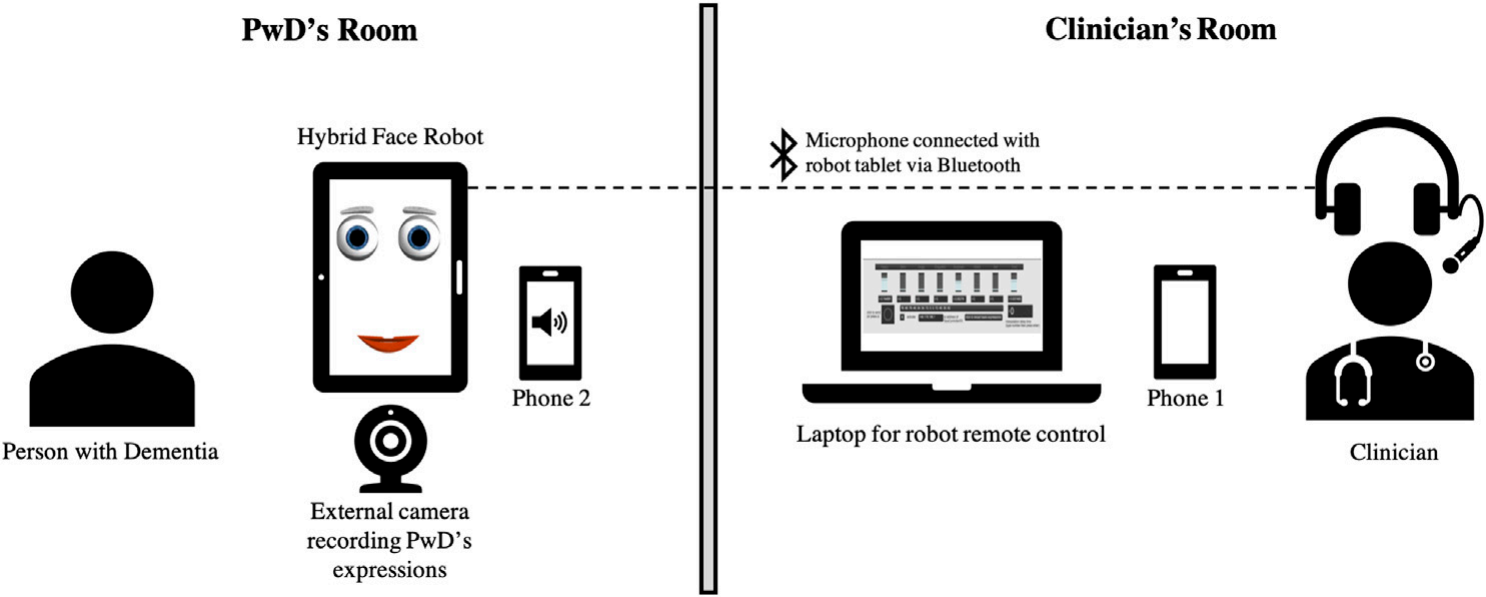
Setup for using the hybrid face robot as a telemedicine interface to deliver cognitive engagement to PwD in the cultural context of South India. PwD’s room includes: tablet with hybrid face robot, webcam to capture participant’s emotions; Clinician’s room includes: laptop with robot’s control, Bluetooth wireless microphone; phone 1 and phone 2 were used to troubleshoot two-way voice communication.

Figure 7 illustrates the experimental setup to conduct remote cognitive engagement using the hybrid face robot, in the form of Wizard of Oz experiments. PwD and clinicians were located in separate rooms to emulate a remote therapy session. PwD was seated in front of the hybrid face robot which was placed on the table. An external webcam was placed right under the robot to record PwD’s expressions and gaze, which will be used for further analysis; video of the whole session was recorded to capture all human-robot interactions. In a separate room, a laptop was used by the clinician to remotely control the robot’s facial expressions (see front-end control in Figure 3). The clinician spoke audio over a Bluetooth wireless microphone connected with the tablet running the robotic face to allow synchronization of the robot’s mouth animation with the clinician’s speech. The two-way verbal communication between PwD and clinician during cognitive engagement sessions took place via an additional phone call connection due to unreliable internet connectivity for smooth voice over IP. The mobile phone in PwD’s room was placed out of sight behind the hybrid face robot as shown in Figure 7 such that participants assumed the voice came from the robot (Wizard of Oz approach). This additional voice connection for two-way communication was required because the robot’s ability to communicate autonomously could not be used, as the regional language in Chennai, India (Tamil) is not yet supported by the IVA. Furthermore, we aimed to provide clinicians with an alternative robotic platform to deliver meaningful cognitive engagement to PwD remotely, which may be of special utility in the COVID-19 era. However, our experimental approach is applicable to other scenarios where in-person meetings with clinicians are not feasible, necessary or desirable.

#### LMIC Pilot Testing in India

This study protocol: “Use of a Hybrid Face Humanoid Robot in Dementia Care: A preliminary study of feasibility and acceptability” was reviewed and approved by the Institutional Ethics Committee (IEC) of the Schizophrenia Research Foundation (SCARF) in Chennai, India. It was executed as a part of ongoing experiments conducted at Dementia Care (DEMCARES), a geriatric outpatient mental health service run by SCARF. All participants were required to provide informed consent before recruitment.

##### Cultural Acceptability and Emotion Recognition in Target Population

The acceptability and cultural appropriateness of the hybrid face robot was explored in South India through qualitative interviewing techniques, such as focus group discussions and in-depth interviews with people with dementia and caregivers, professionals with experience in dementia care, and robotics researchers. We present a user-centred procedure for successful introduction of the new affective robot in different cultures, which involves iterative adjustments based on a set of user studies with healthy older adults and people diagnosed with dementia. We further validate the robot and its facial representation of emotions specifically in the cultural context of South India.

We conducted a series of emotion recognition tasks with a total of N = 14 PwD and N = 26 healthy older adults to assess cultural appropriateness and recognition of robotic facial expressions in South India. Two types of emotion recognition tasks were used. Participants were first shown a sequence of culturally validated pictures of Indian people displaying facial expressions (Mandal, 1987), corresponding to those simulated by the hybrid face robot (see Figure 4). After each emotion observed, participants selected the best match from the given list, following a forced-choice paradigm. Then, the same procedure was followed using the animated facial expressions displayed by the hybrid face robot. The recognition of robot’s emotive responses was compared with the recognition of validated Indian photographs of the corresponding emotions for the two cohorts in India: healthy older citizens and people diagnosed with dementia.

##### Application of Hybrid Face Robot in Dementia Care

Finally, beyond use for social engagement with older adults, we wish to target feasibility of using the robotic platform as a telemedicine system to support mental health, dementia care, and eventually therapeutic intervention. As a basis for this utility, three repeated sessions of remote cognitive engagement using the hybrid face robot, of 30 min each, were conducted with a person with dementia [67 years, male, vascular dementia diagnosis, CDR rating 1 (mild) (Morris, 1991)] at SCARF in Chennai, India. The aim was twofold:

(1) Exploration of the feasibility of such robotic system to be used for cognitive engagement with PwD with regard to end-user acceptance and clinician ease-of-use.
(2) Test system infrastructure in clinical settings to troubleshoot potential technical problems, prior to planned trials and larger scale deployment beyond the COVID-19 pandemic.

The test infrastructure (Section *Designing Infrastructure*) was applied in a set of three robot-assisted cognitive engagement sessions to a person with dementia using the telemedicine interface. Beyond demonstrating the feasibility of the clinician-robot-patient interface in telemedicine for mental health in LMIC setting, we wish to generate initial data on the hybrid face robot as an engagement tool with PwD in the cultural context of South India. A Wizard of Oz approach was used during the three sessions. Hence, a clinician located in a separate room controlled the robot’s range of facial expressions and spoke with the participant ‘through’ the robot (Figure 7). In a separate room, the participant was seated in front of the hybrid face robot. Interactions between the robot and participant included: presentation of the robot, discussion of newspaper articles, and listening to music. The following pre- and post-measures were used to understand the effect of robot-assisted cognitive engagement sessions on mood and engagement, respectively: the face scale (7-item modified version) (Lorish and Maisiak, 1986; Wada et al., 2005b) and the observational measure of engagement (OME) modified (Cohen-Mansfield et al., 2009), a tool to assess direct observations of engagement in people with dementia. The measures were observed by a trained nursing assistant who was present with the participant during experiments. This follows the standard technique described in (Cohen-Mansfield et al., 2009). Repeated sessions were used to acclimatize the participant to the robotic system and develop a level of familiarity. Together with the pre- and post-measures, this enabled a comprehensive comparison of user behavior and engagement in repeated human-robot sessions. After each session, qualitative feedback was collected from the person with dementia, the nursing assistant, caregiver, and clinician.

## Results

### Findings of Human-Robot Interactions with Healthy Participants

#### Emotion Recognition

Table 2 shows the results obtained in a confusion matrix of expression recognition accuracies. The values on each row represent, for a single facial expression, the percentage of responses of the forced-choice. Results showed improved recognition rates compared to our past experiments [see (Bazo et al., 2010; Wairagkar et al., 2020)] All facial expressions showed high recognition rates above 70%. Participants perfectly identified the emotions for happy and tired. Disgusted and stern showed the lowest recognition rates (73.3%) and were often confused between one another, which is in line with our past results. Notably, the recognition rate for disgusted rose from 18.4% (Bazo et al., 2010) to 73.3%. To a lesser degree, afraid was slightly confused with surprised and sad. Afraid shared the elevated eyebrows and opened eyelids of surprise, as well as the sparse downward-curving mouth of sad, which may explain the confusion. Nevertheless, recognition rates were high. In particular, afraid showed an increase to approximately double the recognition rate previously obtained, from 44% (Bazo et al., 2010) to 86.7%.

**TABLE 2:** Expression confusion matrix for the hybrid face robot (% of total per presented expression). Bolded values indicate the % of correctly identified emotions.

	Happy	Stern	Angry	Disgusted	Surprised	Afraid	Sad	Tired
Happy	**100**	0	0	0	0	0	0	0
Stern	0	**73.3**	0	20	0	0	0	6.7
Angry	0	0	**93.3**	6.7	0	0	0	0
Disgusted	0	20	6.7	**73.3**	0	0	0	0
Surprised	0	0	0	0	**93.3**	6.7	0	0
Afraid	0	0	0	0	6.7	**86.7**	6.7	0
Sad	0	0	0	0	0	6.7	**93.3**	0
Tired	0	0	0	0	0	0	0	**100**

Qualitative data from interviews indicates the need to adjust some features for the happy expression to increase its likeability, namely increase the eyebrows’ vertical height (B_hl_ and B_hr_) and show an open mouth (increase M_t_ and M_b_). Though the tired expression showed 100% recognition, participants reported some confusion with stern and disgusted, which can be due to their similarity of partially-closed eyelids and low eyebrows. Despite the added human features, participants’ feedback indicated the addition of the faceplate was perceived unfavourably and the physical depth was poorly perceived. The majority (13 out of 15 participants) disliked the robot and its capacity to show facial expressions when the faceplate was added. We hypothesize our experiments lie on the downslope of the uncanny valley; at this negative gradient, an increase in human likeness (e.g., added faceplate) worsens the human response toward the robot as its partial human appearance moves toward the minimum in the valley. Adding the 3D faceplate to the robot’s digital face appears in this context to induce a stronger uncanny valley effect, which we have considered in subsequent human-robot experiments.

#### User Experience of Multimodal Human-Robot Interactions

**FIGURE 8:**
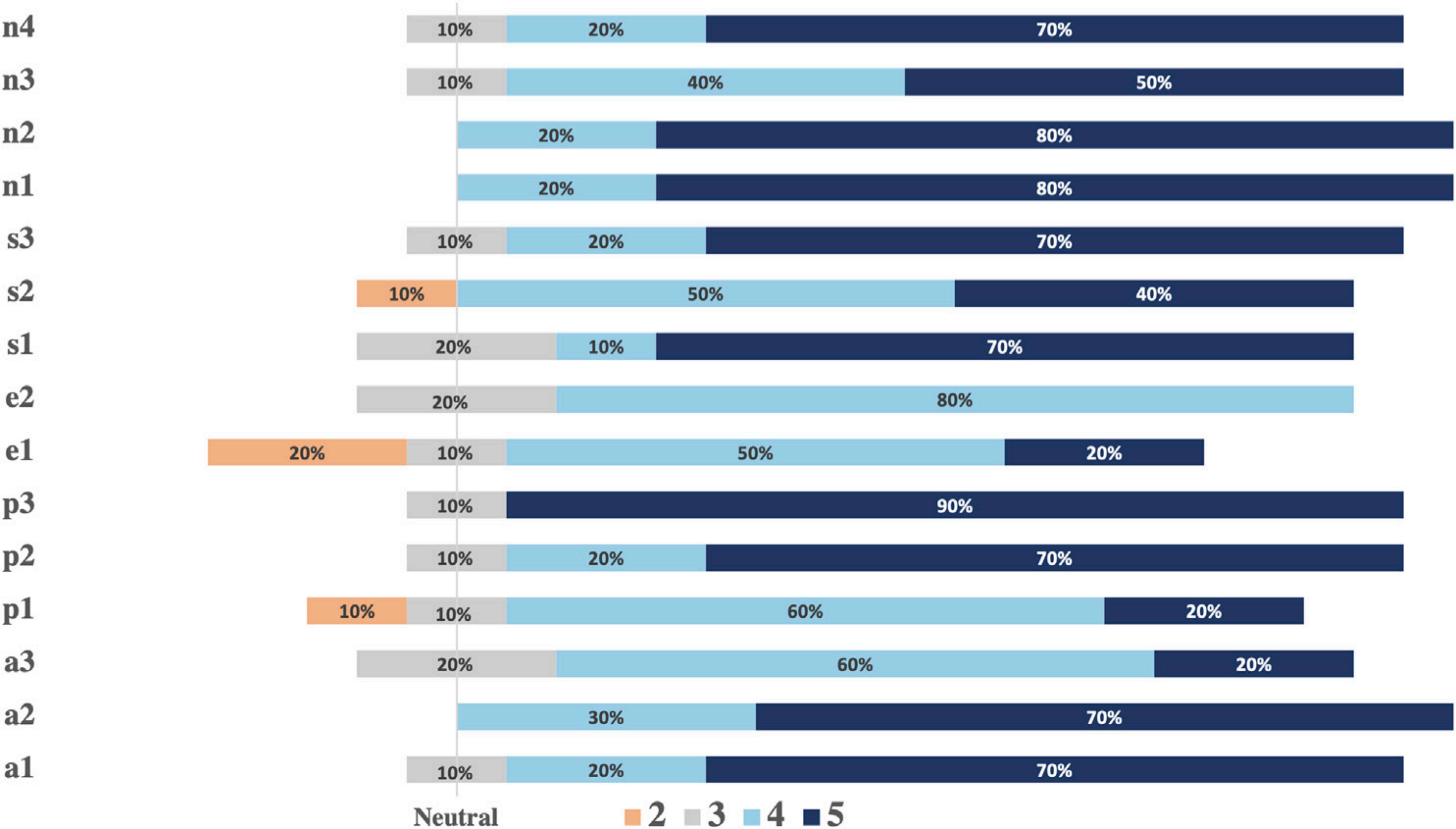
Analysis of the user experience questionnaire. The vertical line marks neutral answers. All positive answers of the 5-point Likert scale are shown on the right with correspondent %. Negative answers and each % are shown on the left side.

Results from UEQ analysis are shown in Figure 8. An overall positive impression of the multimodal robotic system by healthy participants is shown. On average, the novelty and stimulation aspects have received the most positive ratings. Notably, all respondents consistently considered the robot as a creative and inventive robotic platform (n1 and n2) and showed enthusiasm in having new interactions with the robot (s3). The attractiveness aspect showed positive responses. Particularly, 70% of respondents found the possibility to interact with voice extremely useful (a2). The lowest rate within this aspect corresponded to the robot’s design (a3 with 20% neutral answers), which may suggest that our design choice of a simplistic robotic face (with only four facial features) is not sufficient to elicit trust and acceptability in HRI. Regarding questions about perspicuity, there was an overall easiness in interacting and getting familiar with the robot and 90% of participants found the robotic platform easy to use (p3). The efficiency aspect showed the less positive answers (e1, e2) and 20% of participants claimed difficulty identifying the emotion conveyed by the combination of robotic speech and facial expression. In post experimental interviews, all 10 participants reported a strong preference for the multimodal system over pure voice communication, which turned interactions and the overall user experience more “enthusiastic”. Particularly, the robot’s ability to adapt its animated facial representation of emotion in response to user speech in real-time stood out.

### Cross-Cultural Pilot Testing

#### Cultural Acceptability and Emotion Recognition

We have investigated cultural acceptability and feasibility of using the hybrid face robot to support dementia care in India. The overall perception of stakeholders was positive. The preliminary indications are promising as the robot was considered a viable, low-cost and culturally appropriate tool to assist in clinical cognitive engagement with healthy older adults (healthy control—“HC”) and PwD in India. Stakeholders concurred that the possibility of using a hybrid face robot for remote cognitive engagement can potentially help in meaningful engagement of people with dementia at home and also alleviate the perils of social isolation. Plans to deploy the robot as a remote platform to deliver regular cognitive engagement with more participants are underway. Ultimately, these findings suggest the use of this mechanically simple robotic platform for remote cognitive engagement may enhance mental health care and mitigate the impact of COVID-19 on people with dementia.

Emotion recognition experiments with healthy older adults and PwD aimed to assess cultural appropriateness of the hybrid face robot’s simulated facial expressions. Recognition rates of human pictures were similarly high for PwD and HC for all emotions except for afraid, which was well recognized by HC but showed an accuracy below 50% for PwD. We found accuracies for all robot’s simulated facial expressions were lower than expected (between 14 and 62%), with similar values for both PwD and HC. Afraid, angry and disgusted expressions showed the lowest accuracies (between 14 and 20%) for both testing cohorts. Emotions with subtle differences such as surprised and afraid were regularly transposed in user perception. The major difference between the two control groups was observed for the happy expression: double recognition value was obtained for HC. Hence, a discrepancy was observed between cultural recognition of human emotions vs. robotic simulated expressions, however both PwD and healthy older participants perceived the robot’s facial representation of emotion comparably. Results from recognition of robot’s simulated emotions in Chennai, India are in contrast to the high emotion recognition rates obtained with healthy, younger participants in the United Kingdom (Section *Emotion Recognition*). Recognition may show differences due to age, cultural context, and cognitive state. Hence, expressions of the hybrid face robot should be adapted accordingly for use in South India to maximize long-term user engagement and compliance.

#### Pilot Testing Remote Cognitive Engagement for Dementia Support

We piloted repeated sessions with one person diagnosed with dementia as a test of the infrastructure to deliver cognitive engagement in regular sessions. We investigated feasibility of using a mechanically simple, low-cost robotic platform as a robotic telemedicine system to support mental health, particularly in the cultural context of South India. The participant enjoyed interacting with the robot in all three sessions and his mood was rated “very happy” on the pre and post measures of the modified face scale before and after each session, respectively. As shown in Table 3, results from the OME showed a trend of longer duration of engagement with the robot from session 1 (9 min 35 s) to session 3 (18 min 1 s). The participant had no difficulty talking to the robot as assessed by OME “Talking to robot” measure, which received the highest possible score of engagement in all three sessions. Furthermore, the participant was never disruptive during any of the sessions as shown by the lowest possible score received on OME “Disruptive” measure (Table 3).

**TABLE 3:** Results from the observational measure of engagement (OME) modified, which considered the following parameters to assess engagement during robot-assisted clinical sessions: participant’s *attention* on a scale of 1 (very disruptive) to 7 (very attentive); *attitude* to stimulus rated on a scale of 1 (very negative) to 7 (very positive); *duration of engagement* (time until not interested); frequency rate, 0 (none) to 3 (most or all the session), of *talking to the robot*, *talking about the robot* with the nursing assistant, being *disruptive* or *distracted*. The measures were observed by a trained nursing assistant.

Measure		Session 1	Session 2	Session 3
Attention	Average	7	6	5
	Highest	7	7	7
Attitude	Average	4	4	3
	Highest	6	4	4
Duration of engagement		9 min 35 s	10 min 47 s	18 min 1 s
Talking to the robot		3	3	3
Talking about the robot		0	0	0
Disruptive		0	0	0
Distracted		0	1	2

We identified the main areas of technical difficulties and potential improvements for the next planned trials: 1) network connection, which resulted in lags and distortion of voice during remote human-robot sessions; 2) dependency of same network for clinician and PwD; 3) problems in streaming music; 4) lack of a synthetically generated robot’s voice instead of a recognizable human one, which may interfere with participants’ acceptability of the robot. As observed by the nursing assistant, these technical issues often resulted in distraction, impacting participant’s engagement, yet the advantages of robot-assisted cognitive engagement with PwD were acknowledged. The caregiver reported a positive impression of using the hybrid face robot as a telemedicine tool for cognitive engagement and perceived it as a technology to help with her husband’s condition. The clinicians who conducted cognitive engagement sessions commented that the use of affective robotic platforms for engaging persons with dementia, who otherwise are unable to participate in many activities due to the restrictions imposed by pandemic scenarios, holds significant promise. Further work will be necessary to identify factors that will facilitate the use of robotic platforms as a means of telemedicine and develop methods to overcome potential barriers.

Overall, our results demonstrate remote cognitive engagement is feasible with PwD in India using the hybrid face robot as a telemedicine tool. The user-centred design and testing procedure followed with 14 PwD and 26 older adults interacting with the robot, in addition to repeated trials of remote cognitive engagement with one PwD through repeated sessions, provides a basis for deployment with larger participant cohorts. The robotic system may be used as an alternative platform to assist clinicians and support dementia care, which may be especially useful in times when social and medical support of PwD is limited, such as during and beyond the COVID-19 pandemic.

## Discussion

We have introduced a multimodal affective robotic framework to enhance engagement in HRI with capacity to deliver robotic telemedicine to support mental health and dementia care during and beyond the COVID-19 context. We summarize the main findings of the study, their implication for future research and larger scale telemedicine deployment. We also outline the limitations of our investigation and highlight future directions.

### Summary of Findings

At a time of unprecedented overwhelming of global health systems in face of the COVID-19 outbreak, limited social and medical support is delivered to older adults and people living with dementia, who face greater isolation than ever before. Social robots hold significant promise to support mental health and may provide end-users with complementary assistance to stimulate interaction, alleviate anxiety and loneliness, in addition to reducing the caregiver burden, which is a critical need during and after the COVID-19 context. While user trust, complexity and expense of socially assistive robots is a challenge in any setting, we believe there is a larger gap of both resources and targeted research in LMICs. Cultural differences—which influence compliance—as well as technical challenges and cost need to be addressed. In this work, we make progress toward these challenges. In summary, contributions of this investigation include: 1) the robot’s software design optimization; 2) emotion modeling; 3) integration of autonomous conversation capability; 4) testing of the multimodal robotic system with healthy participants in the United Kingdom; 5) validation of the modified robot and its telemedicine interface with older adults with and without dementia in the cultural context of South India.

Our study demonstrates feasibility and cultural appropriateness of robotic telemedicine for mental health support in India. One of the major findings of our study is that cultural adaptation of a social robot is critical—we propose a user-centred procedure that may be followed for successful introduction of a new affective robot in different cultural backgrounds, which involves iterative adjustments based on a set of validation experiments with target users (Section *Robotic Telemedicine for Mental Health Support*). The user-centred procedure followed with 14 PwD and 26 healthy older adults interacting with the robot in South India, in addition to a set of repeated cognitive engagement pilot sessions with one person with dementia, provides a strong foundation for subsequent clinical use.

Our approach for robotic affective communication offers novelty in its mechanically simple, low-cost and multimodal design. We propose it as clinically useful and culturally appropriate technology to deliver cognitive engagement for dementia support in LMICs, particularly in India. Therefore, this social robotic platform may result in a potential telemedicine solution for mental health support of vulnerable populations, not only in the COVID-19 era—which presents a unique opportunity to introduce the robotic system, bringing familiarity with the technology, which may enhance acceptability and compliance in the near future—but also in scenarios where in-person patient-clinician sessions are not logistically feasible or desirable.

### Design Implication and Cultural Adaptation

We optimized the software design and control system of a hybrid face robot comprising an animated digital face that simulates facial expressions based on mathematical affect space emotion mapping with a 3D faceplate to convey realism and depth. This led to considerably higher emotion recognition accuracies than earlier implementations of this style of robot (Section *Emotion Recognition*). More specifically, accuracies above 90% were obtained for happy, tired, sad, angry, surprised and stern/disgusted robotic simulated facial expressions. When separated, stern and disgusted were occasionally mistaken for one another (70%+ accuracy overall) but were easily distinguishable from all other simulated emotions. Furthermore, we have ported the entire robotic system to an inexpensive tablet platform. This highlights the flexibility and adaptability in design of the hybrid face robot, which we have identified as a key feature for cultural usefulness in India. By integrating the robot’s facial expressions with an autonomous conversational engine, we demonstrated real-time adaptable emotion of the robot in response to users’ speech in HRI experiments with healthy participants in the United Kingdom. Although participants did not interact with the robot with different modalities (i.e., speech with and without integration of the robotic expressive face), there was a strong user preference for multimodal over pure voice communication.

To understand the cultural differences in recognition of robot’s simulated emotions, we conducted a series of expression recognition tasks with PwD and healthy older citizens in South India. Despite the increased recognition accuracies obtained from younger participants in the United Kingdom, we observed lower recognition rates for all facial expressions simulated by the robot in India. One potential explanation is the fact that young participants might be more familiar with robotic faces and digital characters, such as emoticons, than older adults tested in India. These findings are very intriguing as a basis for direct comparison between cultural perception of affective emotion. Testing on a wider cohort with parallel controls on subject age, experience with interactive technologies, and possibly education represents a very promising area for future work. In our experiments, we further observed differences between healthy older adults and PwD in India were marginal except for the happy expression, for which double recognition was obtained for the healthy control group. These results inform the cultural acceptability of the robot. As PwD are from the same culture, this allows us to infer that problems with acceptability are unlikely due to the cultural influences but rather due to the effects of cognitive impairment in dementia. Regardless of the current precision of facial expression recognition, stakeholders were positively disposed toward using the robot.

Although further investigation is needed, these studies suggest that emotion recognition of affective robots and the overall effectiveness of HRI are influenced by culture, age, cognitive ability and familiarity with similar technologies. We argue the expressiveness of social robots must be adapted to the culture they will be deployed to following a user-centred and iterative approach to ensure effectiveness, user acceptability and compliance.

### Robotic Telemedicine Beyond COVID-19

While our investigation of human-robot multimodal interactions with healthy participants in the United Kingdom yielded promising results, the AI voice system would need to support regional languages for fully autonomous use in India. Hence, we have designed an alternative test infrastructure to deploy the hybrid face robot as a telemedicine interface to deliver cognitive engagement in regular sessions (Section *Designing Infrastructure*). In our set of user-centred validation studies, including focus group discussions with stakeholders and emotion recognition experiments with 14 PwD and 26 healthy older adults, we identified the hybrid face robot as a feasible, culturally appropriate, and low-cost telemedicine system to support mental health in India. Additionally, we have piloted repeated sessions with one person with dementia as a test of the infrastructure to deliver cognitive engagement in regular sessions. Finally, we have proposed a protocol to introduce the robot for use with PwD and acclimatize the participant to the robot which, through repeated sessions, was received favourably by the participant in experiments, paving the way for further use.

We argue remote cognitive engagement assisted by such robotic platform is feasible with PwD in the cultural context of South India. We observed a trend of increased duration of engagement with the robot from the first to last session, and no alterations in PwD’s mood before and after each session. Positive feedback was obtained from the caregiver and clinician present in robot-assisted sessions. Particularly, the clinician indicated strong promise in using social robotic platforms as a means of telemedicine for dementia support; the caregiver also perceived the robot as a technological tool to help with her husband’s condition. As no similar study has been previously conducted in the literature, this work may provide useful insights into testing and adjusting a hybrid social robot for cognitive engagement with PwD in the cultural context of India and lay the foundation for future telemedicine deployment. This technology may be of special utility, but not limited to dementia support in the COVID-19 era. While the system could be used for other psychological disorders, we wish to establish some veracity through dementia and mental health support of older adults, who are facing more isolation than ever before.

### Limitations and Future Work

Limitations of the AI conversational system integration were acknowledged pointing toward the need for a more natural and unstructured dialogue, and adaptation for mental health applications, e.g., to guide cognitive stimulation therapies for older individuals and PwD. One potential way of increasing trust and acceptability of the AI voice system among the target population is to include different voices and speaking styles. Future improvements of the system architecture should include more training data, i.e., intents, entities and context variables, in the attempt to step beyond a conversational flow. This is a common drawback of existing dialogue systems (Fitzpatrick et al., 2017; Harms et al., 2018). Nevertheless, great efforts are being made in this promising research field to create natural ‘human-like’ conversations (Harms et al., 2018; Griol et al., 2019)^6^, including the exploration of conversational robots and voice-based systems for supporting cognitive impaired individuals (Cruz-Sandoval et al., 2020; Pou-Prom et al., 2020; Salichs et al., 2020). A possibility for future work is to use the camera of the tablet PC running the robot’s software to automatically recognize user emotions. A thorough analysis combining emotion detected from camera, speech and natural language processing could ultimately allow the robot to sense the user’s mood, behavior and personality traits and adapt its response (verbal and nonverbal) in the most appropriate way based on that multimodal feedback, in real-time. Future studies may also use machine learning to adapt behavior to each user over time, which is key for long-term compliance.

One fundamental limitation of the pilot study conducted in India using the robotic telemedicine interface was that only one person with dementia participated. These experiments were logistically very challenging; one of the major drawbacks identified was the screening of patients due to the resources and time available. The main limitation of the experimental setup created for remote cognitive engagement (Section *Designing Infrastructure*) is that both PwD and clinician are required to be connected to the same internet network. We identified lags and distortion of voice during remote clinical sessions as the main technical issue to solve for next trials, in order to ensure maximum engagement. Although the nursing assistant indicated distraction of PwD when technical issues occurred, the participant was overall attentive and enjoyed interacting with the robot. Future experiments could quantify engagement with the robot, with more participants, different types and stages of dementia. Furthermore, for a more direct and rigorous assessment of cultural differences in recognition of robot’s simulated emotions, stricter controls would be needed for subject age, experience with interactive technologies, and cognitive ability. Education level of participants, which may impact acceptability and compliance, could also be used as a metric to be monitored in larger testing cohorts. Future clinical trials and wider deployment could also include end-user training sessions to fully judge the system capability, which may improve its performance.

Finally, aiming to improve robustness, ease of use, availability and scalability of the current system, we have also developed a mobile app working in a similar fashion to the hybrid face robot, as a digital affective robotic platform. Our new mobile-based face robot will allow communication between clinicians and PwD via mobile, without restrictions on location. Even in its current form, our robotic framework provides a more accessible tool to deliver cognitive engagement in LMICs, with potential for positive impact in mental health and dementia care, during and beyond the COVID-19 pandemic. Plans for deployment in India are underway, specifically through robot-assisted telemedicine sessions with older adults and PwD.

## Conclusion

The major contributions of this paper are the development, implementation and pilot testing of a multimodal robotic framework that emotionally interacts through facial expressions and speech to enhance engagement in human-robot interactions. We qualitatively identified the benefits in user engagement of multimodal vs. pure voice communication. We modified this robot further to provide clinicians with a telemedicine interface to deliver regular cognitive engagement, which may be of great utility during and beyond the COVID-19 era. We followed a user-centred design of the robot to ensure it meets the cost constraints and ease of use demands for utility in LMICs, in addition to cultural acceptability. We found cultural validation of a social robot is paramount and introduced a procedure that may inform future studies for engaging human-robot interactions in local cultures. We successfully introduced the modified hybrid face robot into practice for dementia support in LMICs through a pilot study. Results revealed robot-assisted cognitive engagement sessions are feasible in India (and more broadly LMICs), and a trend of longer duration of engagement with the robot was observed through our protocol to introduce the robot to people with dementia in the cultural context of South India. Moreover, clinicians, PwD and caregivers indicated strong promise in the use of this social robotic platform as a means of telemedicine for dementia support in India. Hence, we propose it as an alternative or complementary technological solution to deliver cognitive engagement and enhanced mental health support to older citizens or PwD, during and beyond COVID-19. Plans for deployment in telemedicine sessions specifically motivated by the pandemic are currently underway.

## Data Availability

The original contributions presented in the study are included in the article, further inquiries can be directed to the corresponding author.

## Footnotes

1
https://www.dementiastatistics.org/statistics/hospitals/

2
https://www.alz.co.uk/research/WorldAlzheimerReport2019.pdf

3
https://en.wikipedia.org/wiki/Main_Page

4
https://www.ibm.com/weather

5
https://cloud.ibm.com/docs/assistant?topic=assistant-expression-language

6
https://www.research.ibm.com/artificial-intelligence/project-debater/
